# Correction: Population movement can sustain STI prevalence in remote Australian indigenous communities

**DOI:** 10.1186/1471-2334-14-86

**Published:** 2014-02-19

**Authors:** Ben B Hui, Richard T Gray, David P Wilson, James Ward, Anthony M A Smith, David J Philp, Matthew G Law, Jane S Hocking, David G Regan

**Affiliations:** 1 The Kirby Institute, University of New South Wales, Sydney, NSW 2052, Australia; 2 Baker IDI Heart and Diabetes Institute, Alice Springs, NT 0871, Australia; 3 Australian Research Centre in Sex, Health and Society, La Trobe University, Melbourne, Victoria 3000, Australia; 4 Centre for Women’s Health, Gender and Society, The University of Melbourne, Carlton, Victoria 3053, Australia

## Correction

After the publication of this article [1] it came to our attention that there is a misspelling of one of the authors’ names. The author appearing in the original article as David J Philip should be David J Philp.

## Pre-publication history

The pre-publication history for this paper can be accessed here:

http://www.biomedcentral.com/1471-2334/14/86/prepub
